# Closed Reduction Nailing or Open Reduction Plating in Unstable Paediatric Forearm Fractures: A Case of Paediatric Forearm Fracture Implant Failure

**DOI:** 10.7759/cureus.66175

**Published:** 2024-08-05

**Authors:** Vinod Nair, Harsh S Kumar, Shivappa Devarmani, Abhishek Nair

**Affiliations:** 1 Orthopaedics, Dr. D.Y. Patil Medical College, Hospital, and Research Centre, Dr. D.Y. Patil Vidyapeeth (Deemed to be University), Pune, IND

**Keywords:** paediatric forearm fracture, nailing, plating, implant failure, refracture

## Abstract

A significant amount of all paediatric fractures are forearm fractures involving the radius, ulnar shaft, or both. As surgical stabilisation lowers the likelihood of re-displacement, surgical intervention is currently recommended over conservative treatment of such fractures involving significant displacement and angulation. Open reduction and plating can better anatomically repair the majority of fractures. Bracing is necessary for the first six to eight weeks after nailing since nailing does not give a rigid fixation. External bracing is generally not necessary for plating. In our facility, paediatric diaphyseal forearm fractures are typically treated using titanium elastic nail system (TENS) nailing. However, there are occasional instances where the primary fracture site refractures after surgery, particularly in diaphyseal forearm fractures involving both bones. Our patient was a 12-year-old boy who had come to our facility with a left forearm radius shaft fracture and ulna shaft plastic deformation. The radius shaft fracture was fixed with TENS nailing, and the ulna shaft plastic deformation was corrected by the three-point bending method. Three months later, the patient came back with a refracture of the radius shaft. TENS nail removal, open reduction, and internal fixation of the radius shaft refracture were done with a plate and screws. Anatomic reduction of forearm fractures, open reduction, and the use of plate fixation enable a more thorough correction of malrotation and restoration of the radial bow, allowing for an early range of motion. Since the TENS nail is not a locking device, there is always some amount of mobility at the fracture site, causing loss of reduction, chances of implant failure, and non-union. So primary plating, especially in cases of forearm fractures, appears to be a better option compared to primary TENS nailing in juvenile patients.

## Introduction

Of all paediatric fractures, 5.4% to 14.9% are forearm fractures involving the radius, ulnar shaft, or both [1]. As surgical stabilisation lowers the likelihood of re-displacement, surgical intervention is currently recommended over conservative treatment of such fractures involving significant displacement and angulation [2]. Most fractures can be repaired better anatomically with open reduction and plating [3]. Bracing is necessary for the first six to eight weeks after nailing since nailing does not give a rigid fixation. External bracing is generally not necessary for plating [4]. In our facility, paediatric diaphyseal forearm fractures are typically treated using titanium elastic nail system (TENS) nailing. However, there are occasional instances where the primary fracture site refractures after surgery, particularly in diaphyseal forearm fractures involving both bones. In comparison to other fractures, forearm shaft fractures in children are more likely to result in refracture [5]. Diaphyseal forearm fractures in children have been observed to have refracture rates between 4% and 8% [6]. Previous studies have highlighted greenstick fractures, persistent angulation, and radiographically immature fracture healing as risk factors for the refracture of paediatric forearm fractures after both surgical and conservative management [7, 8]. Among the specific risk factors for refracture known to exist in individuals who underwent intramedullary nailing are implant removal within six months of fixation, young patients with low body weight, and a smaller bone diameter of the forearm [9]. In this case report, we discuss the management of a paediatric forearm fracture implant failure. We have obtained the consent of the child's parents for the publication of this case report.

## Case presentation

**Figure 1:**
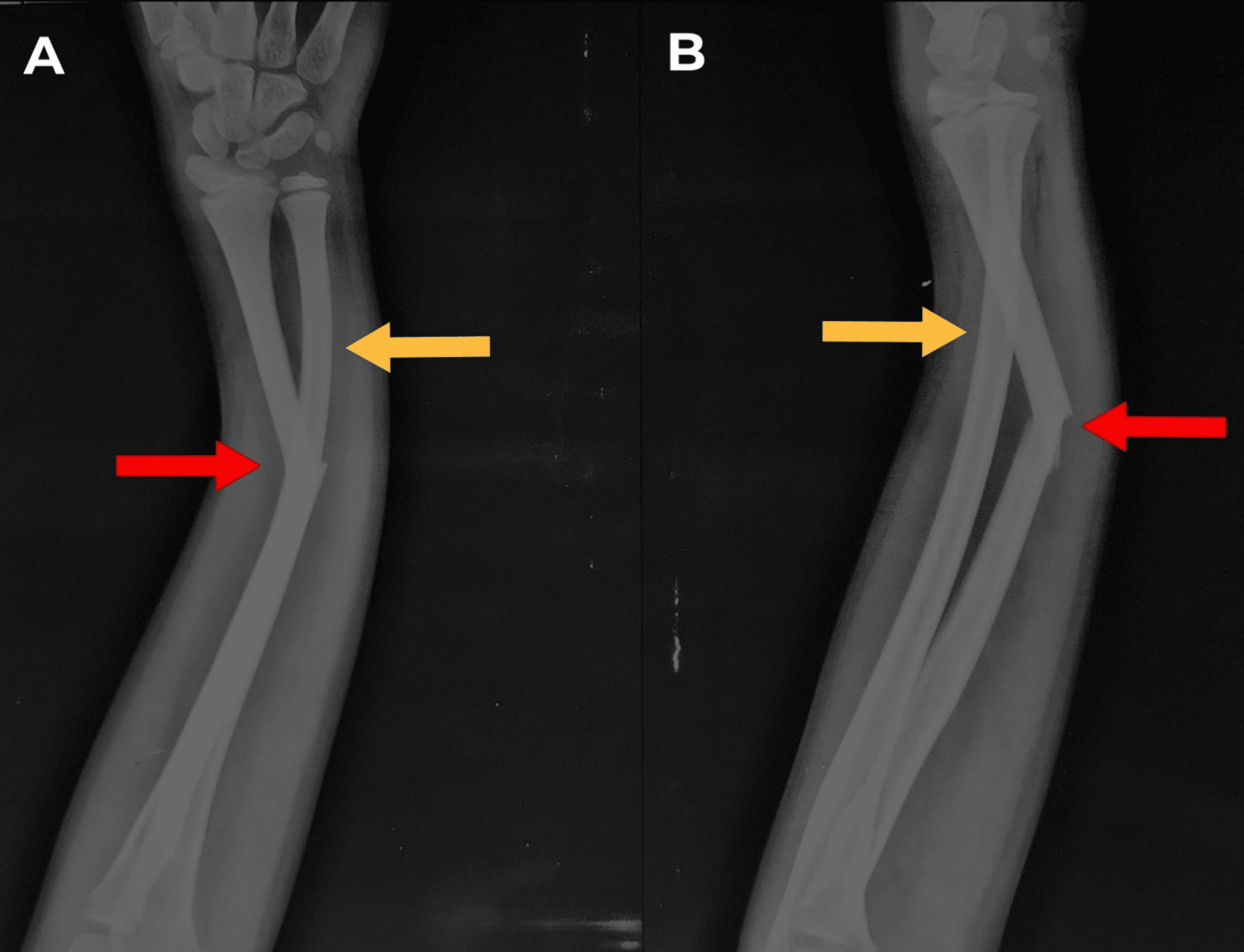
Left forearm full-length AP (A) and lateral (B) view of radius and ulna showing radius shaft fracture (red arrow) and plastic deformation of the ulna (yellow arrow) AP: Anterior posterior

A 12-year-old boy, right-handed, with no prior surgical or medical history, came into our emergency room with complaints of pain and swelling in his left forearm. He had a history of falling onto his left forearm one day prior. Examination of the left forearm showed mild swelling and tenderness over the left forearm radius mid-shaft region, with visible deformity of the left forearm and no open wound. No distal neurovascular impairment was seen. A left forearm full-length X-ray, anterior-posterior (AP) view, and lateral view (Figure 1) showed radius shaft fracture with ulnar and volar angulation, respectively, with plastic deformation of the ulna.

Fracture stabilisation was done with an above-elbow slab. All required pre-operative investigations were completed following the patient's admission to the hospital. The pre-anesthesia evaluation was completed, and clearance for open reduction and intramedullary nailing under general anaesthesia was granted. We explained to his family the complications, such as compartment syndrome, refracture, non-union, and malunion.

**Figure 2:**
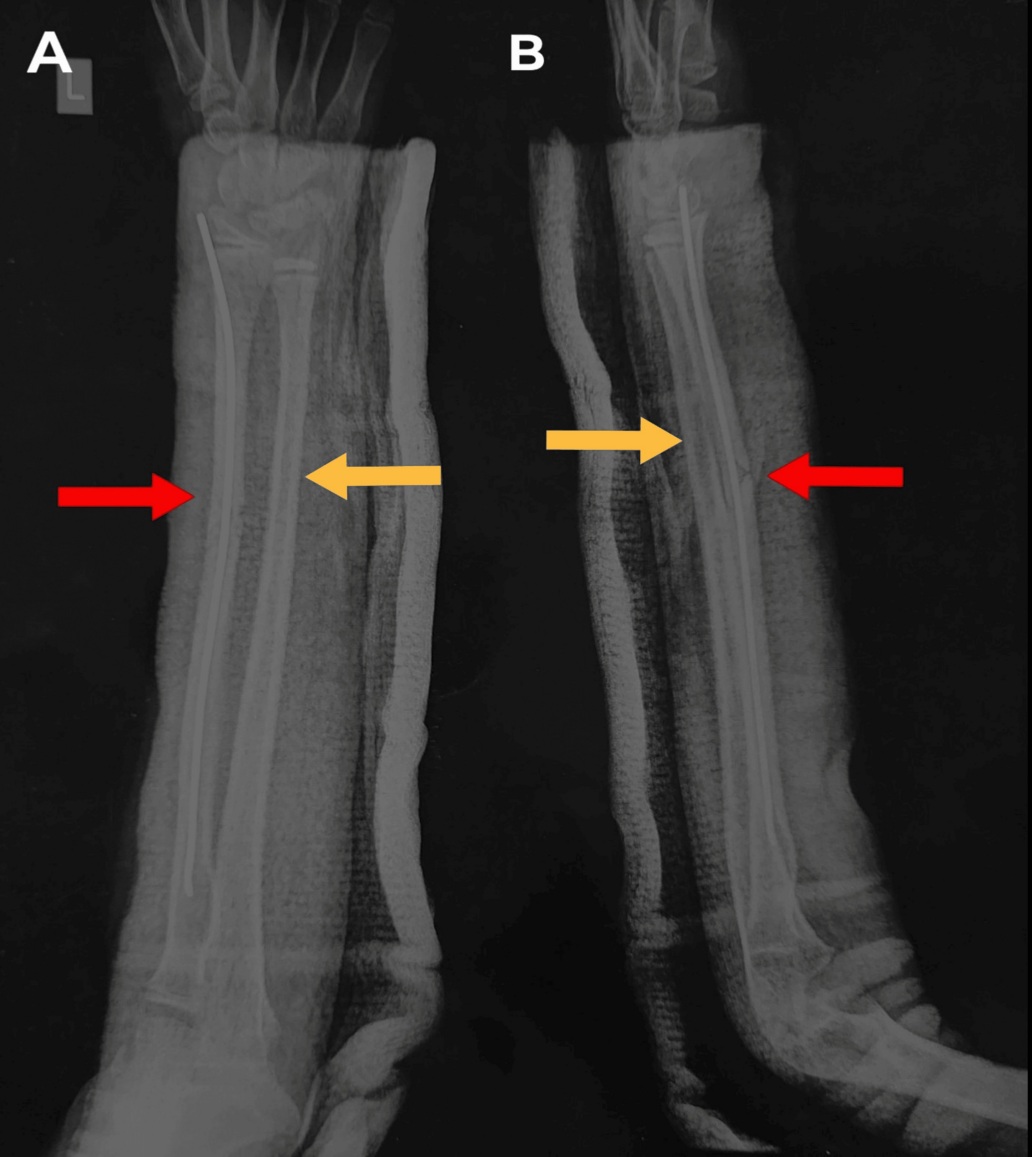
Left forearm full-length AP (A) and lateral (B) view of radius and ulna showing TENS nailing of radius shaft (red arrow) and ulna plastic deformation correction (yellow arrow) AP: Anterior posterior; TENS: titanium elastic nail system

On the day of the surgery, the patient was assessed pre-operatively for general anesthesia. Antibiotics were administered prior to surgery. The patient was taken in a supine position with a side-arm table. Scrubbing, painting, and draping were done. The ulna plastic deformation was reduced with a steady three-point bending method. The radius shaft fracture was reduced by opening the fracture site by Henry’s approach with a 4 cm incision. In the reduced position of the fracture, an intramedullary TENS nail was inserted through the lateral aspect of the distal radius proximal to the physis. The reduction was adequate. An above-elbow slab was applied, and closure was done in the standard manner. Post-operative AP and lateral X-rays (Figure 2) of the left forearm showed the ulna plastic deformation and the radius shaft fracture were properly reduced.

At two and four weeks after surgery, the patient was evaluated. Suture removal was done after a follow-up examination at two weeks. At a follow-up of four weeks, the slab was removed, and wrist and elbow normal range of movements were started. The patient did not come for follow-up after four weeks.

**Figure 3:**
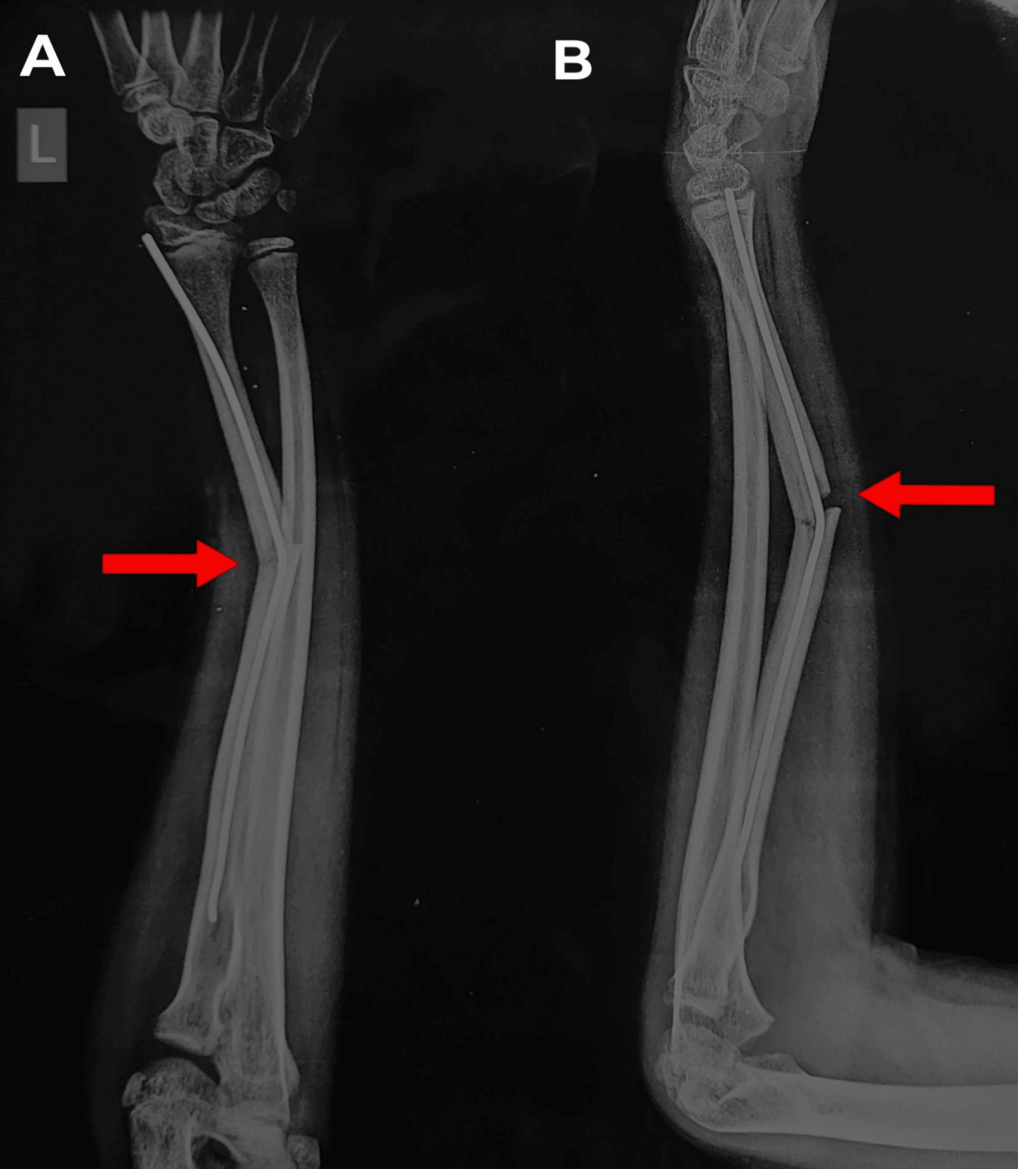
Left forearm full-length AP (A) and lateral (B) view of radius and ulna showing radius shaft refracture and bending of TENS nail (red arrow) AP: Anterior posterior; TENS: titanium elastic nail system

At three months post-operatively, the patient came to our emergency room with pain and swelling over the previously operated left forearm. He had a history of falling onto his left forearm six days ago. The examination of the left forearm showed mild swelling and tenderness over the left forearm radius mid-shaft region, with visible deformity of the left forearm and no open wound. No distal neurovascular impairment was seen. A left forearm full-length X-ray, AP view, and lateral view (Figure 3) showed radius shaft refracture at the primary fracture site with ulnar and volar angulation, respectively, with a bent intramedullary TENS nail.

Following the patient's admission to the hospital, all required pre-operative assessments were completed in the same manner. After a pre-anaesthetic examination, the patient was deemed fit for the intramedullary TENS nail removal, open reduction, and internal fixation with plating procedure under general anaesthesia. The expected intra-op and post-surgical complications were explained to the family as before.

On the day of the surgery, the patient had a pre-operative evaluation for general anesthesia. Antibiotics were given prior to surgery. With a side-arm table, the patient was placed in a supine position. Scrubbing, painting, and draping were done. The bent TENS nail was removed. The fracture was reduced and fixed with a 2.7 mm eight-hole dynamic compression plate (DCP) through Henry’s approach. The reduction was satisfactory, the closure was done in a routine manner, and the above-elbow slab was applied.

**Figure 4:**
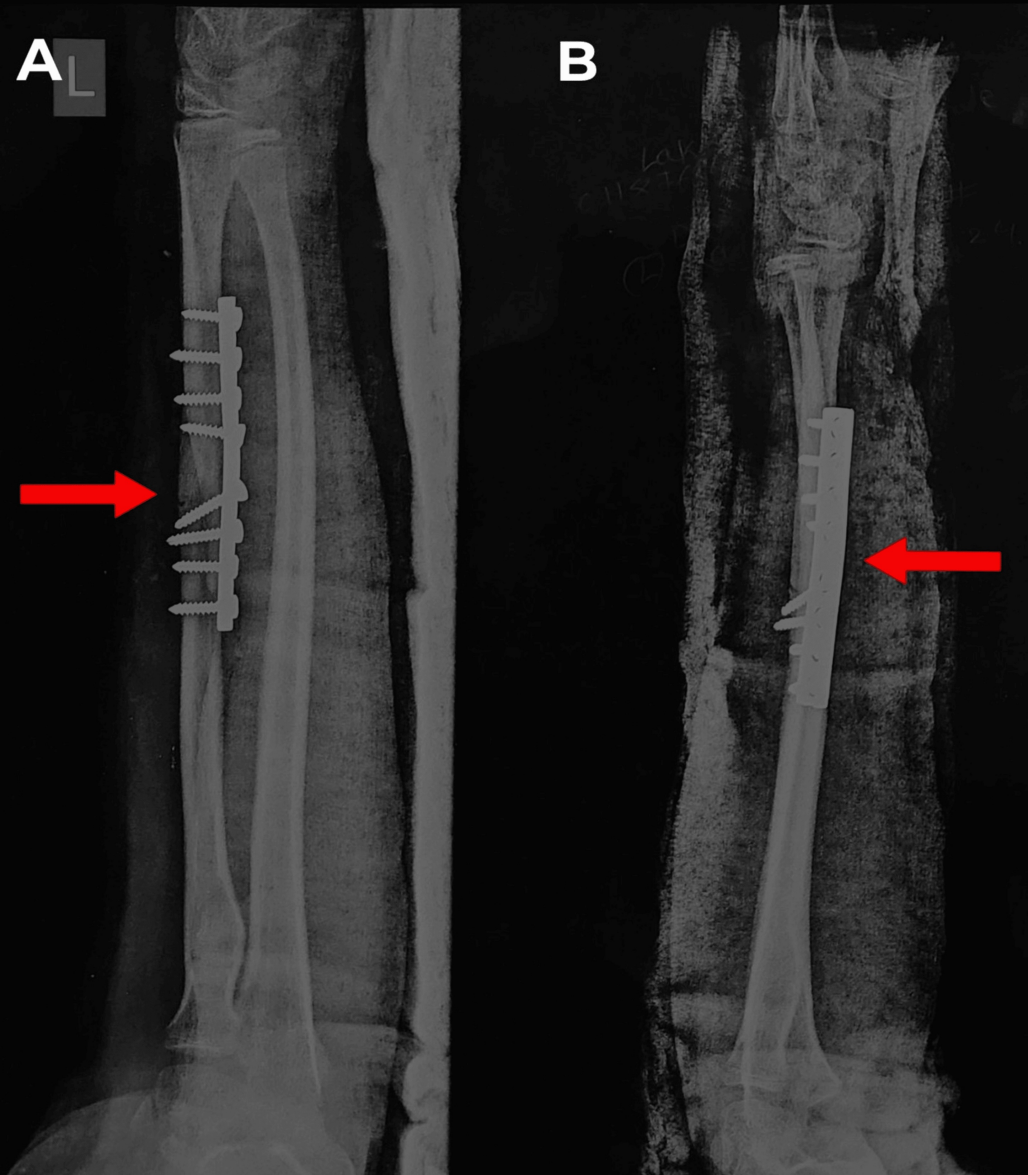
Left forearm full-length AP (A) and lateral (B) view of radius and ulna showing plating of radius shaft refracture (red arrow) AP: Anterior posterior

Post-operative AP and lateral X-rays (Figure 4) of the left forearm showed the radius shaft fracture was properly reduced.

At two and four weeks after surgery, the patient was evaluated. Suture removal was done after a follow-up examination at two weeks. At a follow-up of four weeks, the slab was removed, and wrist and elbow normal range of movements were started. The patient is now able to perform daily activities with a good elbow and wrist range of motion.

## Discussion

Of all paediatric fractures, 5.4% to 14.9% are found to be forearm fractures involving the radius, ulnar shaft, or both [1]. In our case, the patient presented with a radius shaft fracture and ulna plastic deformation, which was managed by intramedullary nailing with a TENS nail for the radius shaft, and the ulna plastic deformation was reduced with a steady three-point bending method. However, a further minor fall resulting in refracture of the radius shaft and an implant failure required an open reduction and internal fixation with a plate. This modality of treatment provided a more rigid fixation compared to the TENS nail with good radiographic and functional recovery.

Perfect anatomic reduction is not required in children under the age of 10 since remodelling may be able to rectify residual deformity [10]. Several investigations have demonstrated that because remodelling is uncertain, forearm angulations higher than 10° should be addressed [11]. According to Fuller, there is a direct correlation between the decrease in rotational malunion and the loss of pronation and supination. He observed that in girls older than eight years old and boys older than 10 years old, there is no spontaneous repair of deformity in cases of malunion [12]. For kids older than 10, maintaining a complete range of motion requires a near-anatomic reduction [10,11]. The impaired function was the result of recurring issues following conservative treatment, such as consolidation in poor alignment and loss of correction requiring repeated reduction. As a result, the way children with unstable forearm fractures were treated was altered [12-14]. 

As our patient was a 12-year-old child with an unstable forearm shaft fracture with a radius shaft angulation greater than 10°, we managed the case with surgical treatment. As surgical stabilisation lowers the likelihood of re-displacement, surgical intervention is currently recommended over conservative treatment of such fractures involving significant displacement and angulation [2]. For the management of children with unstable forearm shaft fractures, numerous surgical management techniques have been documented, including external fixations, plate fixation, pins with plaster, and intramedullary nailing [13,15-17]. We decided to manage our case by internal fixation with an intramedullary TENS nail, as this modality involves limited soft tissue dissection and provides a stable internal fixation.

In comparison to other fractures, forearm shaft fractures in children are more likely to result in refracture [5]. Greenstick fractures, persistent angulation, and radiographically immature fracture healing have all been identified in prior research as risk factors for refracture of paediatric forearm fractures following both conservative and surgical management [7,8]. Some specific risk factors for refracture that are known to exist in individuals who underwent intramedullary nailing include younger patients with low body weight, a smaller bone diameter of the forearm, and the removal of nails within six months of fixation [9]. As seen in this case, a further minor fall resulted in implant failure and refracture of the radius shaft.

Lately, closed methods have proven to be an effective treatment for the majority of both-bone forearm fractures in children aged 10 to 16. Open reduction and internal fixation are required in certain situations, nevertheless, where closed reduction is deemed unsuitable [18, 19]. Schemitsch et al., in a study, cited that the majority of fractures can be repaired better anatomically with open reduction and plating [3]. Bracing is necessary for the first six to eight weeks after nailing since nailing does not give a rigid fixation. External bracing is generally not necessary for plating [4]. Generally, in order to achieve sufficient reduction and proper implant placement, open reduction and internal fixation procedures require exposing the whole fracture. The radial bow may be recreated with this technique, which also provides axial and rotational control over the reduction, two essential components for restoring forearm range of motion and achieving favourable functional results [20]. As the TENS nail fixation in our case provided less rigid fixation than a plate, the refracture of the radius shaft was managed by open reduction and internal fixation with a plate. 

In summary, anatomic reduction of forearm fractures, open reduction, and the use of plate fixation enable a more thorough correction of malrotation and restoration of the radial bow, allowing for early range of motion. Also, due to the 100% reduction and achievement of proper radioulnar interosseous space in the plate compared to the nail, the chances of loss of reduction, malalignment or malunion, and implant failure leading to refracture are decreased with the plate as it gives a more rigid fixation compared to the nail. Since the TENS nail is not a locking device, there is always some amount of mobility at the fracture site, causing loss of reduction, chances of implant failure, and non-union.

## Conclusions

We presented this case to describe a complication of implant failure and refracture due to inadequate strength and stabilisation given by a TENS nail in a forearm fracture in a juvenile patient. This complication was managed by open reduction and internal fixation with plating, which resulted in an excellent radiographic and functional recovery. Thus, primary plating in cases of forearm fractures appears to be a better option compared to primary TENS nailing in juvenile patients, which can prevent such refractures. However, to fully comprehend its efficacy, we will need to continue to follow up and study a larger sample size.
